# Effect of helium pre- or postconditioning on signal transduction kinases in patients undergoing coronary artery bypass graft surgery

**DOI:** 10.1186/s12967-016-1045-z

**Published:** 2016-10-14

**Authors:** Kirsten F. Smit, Daniel Brevoord, Stefan De Hert, Bas A. de Mol, Raphaela P. Kerindongo, Susan van Dieren, Wolfgang S. Schlack, Markus W. Hollmann, Nina C. Weber, Benedikt Preckel

**Affiliations:** 1 Laboratory of Experimental Intensive Care and Anesthesiology (L.E.I.C.A.), Department of Anesthesiology, Academic Medical Centre (AMC), Meibergdreef 9, 1100 DD Amsterdam, The Netherlands; 2 Department of Cardiothoracic Surgery, Academic Medical Centre (AMC), Amsterdam, The Netherlands; 3 Department of Anesthesiology, Ghent University, Ghent, Belgium

**Keywords:** Noble gases, Helium, Preconditioning, Postconditioning, Translation, CABG surgery, P38 MAPK, ERK1/2, HSP-27, PKC-ε

## Abstract

**Background:**

The noble gas helium induces pre- and postconditioning in animals and humans. Volatile anesthetics induce cardioprotection in humans undergoing coronary artery bypass graft (CABG) surgery. We hypothesized that helium induces pre- and postconditioning in CABG-patients, affecting signaling molecules protein kinase C-epsilon (PKC-ε), p38 mitogen activated protein kinase (p38 MAPK), extracellular signal-regulated kinase 1/2 (ERK-1/2) and heat shock protein 27 (HSP-27) within cardiac tissue, and reducing postoperative troponin levels.

**Methods:**

After ethical approval and informed consent, 125 elective patients undergoing CABG surgery were randomised into this prospective, placebo controlled, investigator blinded, parallel arm single-centre study. Helium preconditioning (3 × 5 min of 70 % helium and 30 % oxygen) was applied before aortic cross clamping; postconditioning (15 min of helium) was applied before release of the aortic cross clamp. Signaling molecules were measured in right atrial appendix specimens. Troponin-T was measured at 4, 12, 24 and 48 h postoperatively.

**Results:**

Baseline characteristics of all groups were similar. Helium preconditioning did not significantly alter the primary outcome (molecular levels of kinases PKC-ε and HSP-27, ratio of activated p38 MAPK or ERK ½). Postoperative troponin T was 11 arbitrary units [5, 31; area-under-the-curve (interquartile range)] for controls, and no statistically significant changes were observed after helium preconditioning [He-pre: 11 (6, 18)], helium postconditioning [He-post: 11 (8, 15)], helium pre- and postconditioning [He-PP: 14 (6, 20)] and after sevoflurane preconditioning [APC: 12 (8, 24), p = 0.13]. No adverse effects related to study treatment were observed in this study.

**Conclusions:**

No effect was observed of helium preconditioning, postconditioning or the combination thereof on activation of p38 MAPK, ERK 1/2 or levels of HSP27 and PKC-ε in the human heart. Helium pre- and postconditioning did not affect postoperative troponin release in patients undergoing CABG surgery.

*Clinical trial number* Dutch trial register (http://www.trialregister.nl/) number NTR1226

**Electronic supplementary material:**

The online version of this article (doi:10.1186/s12967-016-1045-z) contains supplementary material, which is available to authorized users.

## Background

Noble gases like xenon can induce cardioprotection via preconditioning [1–6]. The signal transduction cascade mediating this effect has partly been described and shares similarities with transduction cascade mediating ischemic preconditioning [7, 8]. This noble gas induced cardioprotective effect was abolished on a cellular level by blockers of protein kinase C (PKC) and p38 mitogen activated protein kinase (p38 MAPK) [3]. Xenon preconditioning also involves extracellular-signal-regulated kinases-1 and -2 (ERK1/2) [6], leading to intracellular translocation of heat shock protein 27 (HSP-27) [4].

The non-anesthetic noble gas helium has no relevant cardiopulmonary side effects and is already clinically used in patients with airway diseases [9, 10]. It can easily and safely be administered using readily available ventilators and in critical care patients [11, 12]. Experimental data from different laboratories in different animal species have demonstrated profound protective effects of helium against ischemia–reperfusion damage of the heart [1, 13, 14]. In a previous study in healthy volunteers, we demonstrated that 3 times 5 min of 79 % helium inhalation prevented post-ischemic endothelial dysfunction [15]. Experimental data indicated involvement of similar signal cascades during helium conditioning as were shown before for xenon and anesthetic induced conditioning [1]. However, the exact underlying mechanism of helium protection in humans remains unclear.

Different preconditioning protocols are currently used to induce anesthetic preconditioning, either via continuous administration throughout surgery [16], during ischemia/reperfusion [17] or before aortic cross clamping [18]. It is known that both, timing and repetition of the preconditioning stimulus, are central for producing the respective protection.

Based on the experimental and first clinical data on helium conditioning we hypothesized that helium induces pre- and/or postconditioning in human myocardium of patients undergoing CABG surgery, involving regulation of PKC, p38 MAPK, ERK 1/2 and HSP-27, and reducing postoperative troponin T release.

## Methods

**Fig. 1:**
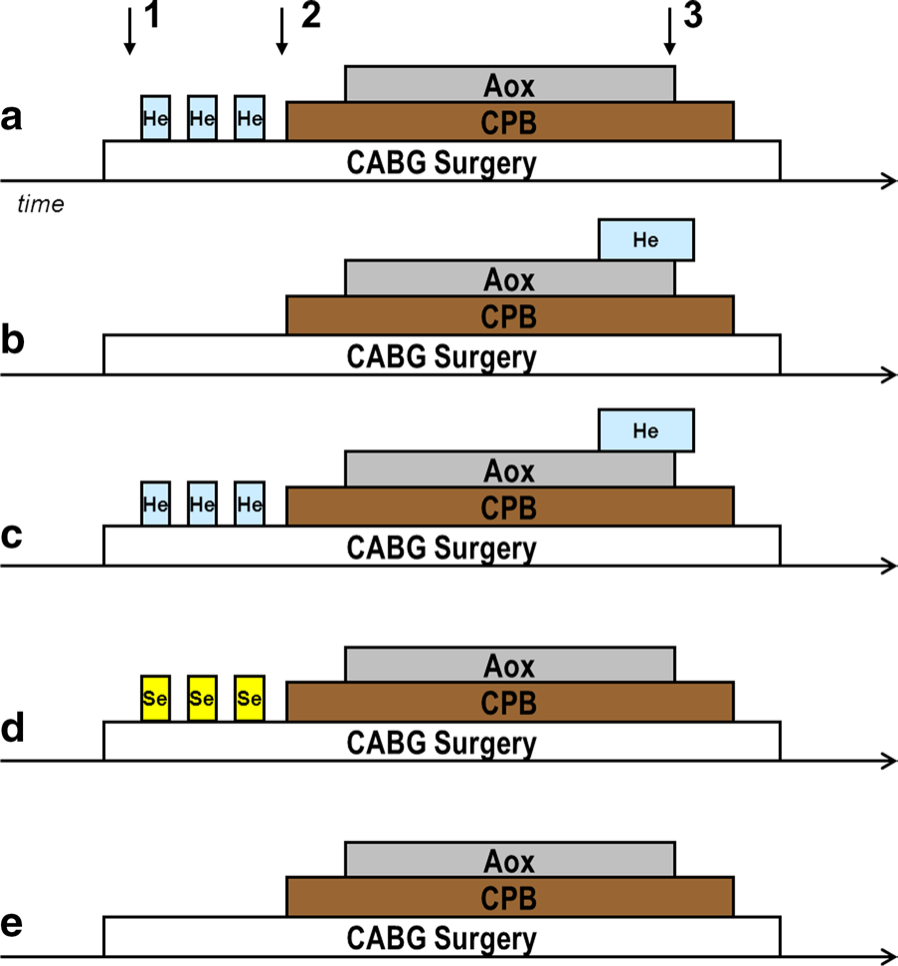
Protocol outline. Schematic timeline of the study protocol. The *black arrows* represent the time points at which atrial myocardial biopsies were taken. Aox: aortic cross clamp. CPB: cardiopulmonary bypass**. a** Helium preconditioning group. Helium was administered in three cycles for 5 min, followed by 5 min inhalation of oxygen enriched air (30 % oxygen). **b** Helium postconditioning group, helium administration started at the end of aortic cross clamping for 15 min and was continued for 5 min after begin of reperfusion. **c** Helium pre-and postconditioning group. Helium was administered both as preconditioning stimulus before cardiopulmonary bypass and as postconditioning stimulus at the end of aortic cross clamping. **d** Anesthetic preconditioning group in which sevoflurane was administered in three cycles of 5 min. **e** Untreated controls

The institutional review board of the Academic Medical Center, Amsterdam, The Netherlands, approved the trial registered in the Dutch trial register (number NTR1226). Inclusion into this prospective, placebo controlled, investigator blinded, parallel arm single-center study took place from 8/7/2008 to 3/7/2011 at the Academic Medical Center, Amsterdam, The Netherlands, in accordance with the International Conference on Harmonization on Good Clinical Practice Guidelines and the Declaration of Helsinki. Patients were randomized to one of five parallel groups in a 1:1:1:1:1 allocation ratio using web-based randomization software (ALEA; NKI; Amsterdam, The Netherlands) with a fixed block scheme, a block size of 5 patients and stratification on sex (see Fig. 1). While the anesthesiologist and the investigator in the operating room were not blinded, the patients as well as the investigators performing laboratory data analysis (troponin T values, Western Blot experiments) were blinded to the randomization strategy. Patients were recruited by self-selecting, and all subjects gave written informed consent. Exclusion criteria were age <18 years, legal incapacity, emergency operations, combined coronary artery and heart valve procedures, off-pump procedures, diabetes mellitus, severe chronic obstructive pulmonary disease (COPD), and left ventricular ejection fraction <30 %. The last two criteria were added after publication on http://www.trialregister.nl but before start of the study.

### Study protocol

At least two cycles inhalation of sevoflurane were necessary to induce preconditioning in humans [18, 19]. We wanted to extend the preconditioning stimulus and decided to use three cycles of conditioning, as this protocol was also used in most experimental studies [20]. The first group received helium preconditioning (He-Pre) by inhalation of three cycles of helium for 5 min, followed by 5 min inhalation of oxygen-enriched air (30 % oxygen). Helium was obtained as a mixture with oxygen (Heliox: 79 % helium and 21 % oxygen, BOC, Mordon, United Kingdom) and administered using a non-invasive helium delivery system (Helontix Vent, Linde Therapeutics, Eindhoven, The Netherlands) modified to allow manual ventilation in a Maplesons A configuration. All patients were ventilated the same way by the same investigator. Extra oxygen was added and the final concentration of the gas-mixture was 70 % helium and 30 % oxygen. He-Pre was administered shortly before start of cardiopulmonary bypass (CPB). A graphical presentation of our study protocol is represented in Fig. 1. The postconditioning group (He-Post) received at least 15 min of helium at the end of aortic cross-clamping, lasting up to 5 min after release of the clamp. The third group received helium as pre- and postconditioning stimulus (He-PP). Patients receiving helium pre- and postconditioning thus received two conditioning stimuli of helium with double time of helium ventilation. To compare the effects of helium with the known effects of anesthetic preconditioning (APC), the fourth group received three cycles of 5 min sevoflurane inhalation with a minimal alveolar concentration (MAC) of 1.0 MAC, and the auto-flow function of the anesthesia machine (Zeus, Dräger Medical, Lübeck, Germany) was used to ensure rapid wash in and wash out of sevoflurane. The 5th group was an untreated control group.

### Anesthesia

Patients received premedication with temazepam 10 mg per os. Induction of anesthesia was performed with intravenous administration of midazolam 0.1–0.2 mg kg^−1^ and target controlled infusion of propofol (dosage was 1–2 mg/kg for induction), sufentanil 1.0–1.5 μg kg^−1^, and rocuronium 0.6 mg kg^−1^ for muscle relaxation. Target controlled infusion of propofol was continued to maintain anesthesia in combination with either continuously or intermittently sufentanil.

### Surgery

All patients received routine monitoring during operation and routine surgical techniques were used. A pulmonary artery catheter was used for cardiac output monitoring. The left internal mammary artery was used to graft the left anterior descending artery. As additional grafts, harvested veins from the leg, the right internal mammary artery or one of the radial arteries were used. Both, cold crystalloid and cold blood cardioplegia were administered antegrade via the aortic root, and management of the cardiopulmonary bypass (CPB) was according to standard procedure.

Median sternotomy was performed, followed by pericardiotomy after which the first sample of the right atrial appendage was obtained. Then the left internal thoracic artery was prepared, during which time systemic heparinization was started (300 IU/kg goal: coagulation time >450 s). After venous and arterial cannulas for CPB were inserted and secured, the second sample of the right atrium was obtained which was directly after preconditioning in the applicable groups. Then CPB was started, and the aorta was cross-clamped and cardioplegia solution was infused. All distal anastomoses were performed during aortic cross-clamping. Additional cardioplegic solution was administered at intervals to maintain a flat electrocardiogram. Fifteen minutes before expected release of the aortic cross clamp, we started helium postconditioning in the designated groups, and continued helium ventilation until 5 min after the start of reperfusion. After completion of coronary artery bypass grafting, CPB was discontinued and the third sample of the right atrium was obtained. After surgery, patients were transferred to the intensive care unit (ICU), received routine therapy and were weaned from the ventilator. ICU and ward staffs were blinded to the treatment allocation.

### Blood sampling and tissue preparation

Blood samples were taken before cardiopulmonary bypass, 10 min after cardiopulmonary bypass and at the end of operation, as well as at 4, 12, 24 and 48 h after cardiopulmonary bypass. We measured troponin-T, creatinine kinase and its myocardial specific isoform Creatine Kinase-Muscle/Brain as markers of cellular injury. All samples were analysed in the Laboratory of Clinical Chemistry of the Academic Medical Centre, Amsterdam, The Netherlands.

Atrial samples were immediately flash frozen in liquid nitrogen and stored at −80 ^◦^C until further processing. Tissue fractionation was performed as described by Weber et al. [6]. Cytosolic, membrane, and the particulate fraction were immunoblotted using the Criterion Western Blotting system (Biorad, Hercules, CA).

After protein determination by the Lowry method, samples were thawed and diluted 1:5 with Sample Buffer 5 times containing Tris–HCl, glycerol and bromophenol blue. Samples were vortexed and boiled at 95 °C before being subjected to sodium dodecyl sulfate–polyacrylamide gel electrophoresis using Criterion™ XT precast gels (Biorad, Hercules, CA). The proteins were separated by electrophoresis and transferred to a polyvinylidenfluorid membrane by tank blotting (Voltage 200 V for 50–55 min). Non-specific binding of the antibody was blocked by incubation with 5 % fat dry milk powder or bovine serum albumin solution in tris-buffered saline containing tween (TBS-T) for 2 h. Subsequently, the membrane was incubated overnight at 4 °C with the respective primary antibody at indicated concentrations. After washing in fresh, cold TBS-T, the blot was subjected to the appropriate horseradish peroxidase-conjugated secondary antibody for 2 h at room temperature. Immunoreactive bands were seen by chemiluminescence detected on X-ray film (Hyperfilm ECL, Amersham) using the enhanced chemiluminescence system Santa Cruz. The blots were quantified using a Kodak Image station^®^ (Eastman Kodak Co., Rochester, NY, USA) and the results are presented as the ratio of phosphorylated to total protein. Values are expressed as *x*-fold average light intensity (AVI) compared with control. Equal loading of protein on the gel was additionally confirmed by detection of actin/α-tubulin and Coomassie staining of the gels.

### Antibodies

We used anti-phospho PKC-ε, antibody (1:10.000) and total PKC-ε, both from Upstate (Lake Placid, NY). Phospho-ERK1/2 (1:10.000), total ERK 1/2 (1:10.000), Phospho p38 MAPK (1:5.000) and total p38 MAPK (1:5.000) were obtained from Cell Signalling (Danvers, MA), HSP 27 (1:5.000) from Abcam (Cambridge, UK). Both actin (1:10.000) and α-tubulin (1:40.000) were obtained from Sigma (St. Louis, MO). Peroxidase-conjugated goat anti-rabbit and donkey anti-mouse antibodies were from Jackson Immunoresearch (Suffolk, UK). The enhanced chemiluminescence protein detection kit was purchased from Santa Cruz (Heidelberg, Germany).

### Endpoints and data collection

Primary endpoints of this study are phosphorylation of ERK1/2, p38MAPK and expression of HSP27 and PKC-ε in the particulate fraction. Secondary endpoints include post-operative troponin T release.

Data were collected on age, sex, race, length and weight, co-morbidities and risk factors for cardiovascular disease, Euroscore, medication usage, duration of bypass, and aortic clamping, number and type of grafts. Because of technical difficulties establishing reproducible results for the western blot we lost n = 5 patients per group for these targets (p38 MAPK, ERK1/2, HSP27 and PKC-ε).

### Sample size calculation and statistics

Regarding our primary endpoint, no data on the effect of noble gas preconditioning on protein expression in human myocardial tissue was available while setting up the study. A proper sample size calculation was therefore not possible at start of the study. However, based on previous experimental research and a similar clinical study [19], we expected to find any—also clinically relevant differences—with a sample size of 25 patients per group.

Numerical data are presented as mean ± SD or median with interquartile range, as appropriate. Categorical data are presented as numbers and percentages. Statistical analyses were done using SPSS version 22 (IBM, Armonk, New York, USA).

We considered a *p* value of <0.05 to be statistically significant. Statistical testing of the western blot data was done using Shapiro–Wilk test for normality and Friedmann test for non-parametric data followed by Bonferroni correction for multiple testing (GraphPad Prism version 5.0, GraphPad, La Jolla, CA). We chose to graphically represent our data as mean + SD, however detailed information regarding the mean differences of the timepoints from our primary endpoints is available in Additional file 1.

To compare post-operative troponin T release the area-under-the-curve was calculated (mentioned as arbitrary units) and compared in a one-way-ANOVA.

## Results

### Baseline characteristics

**Fig. 2:**
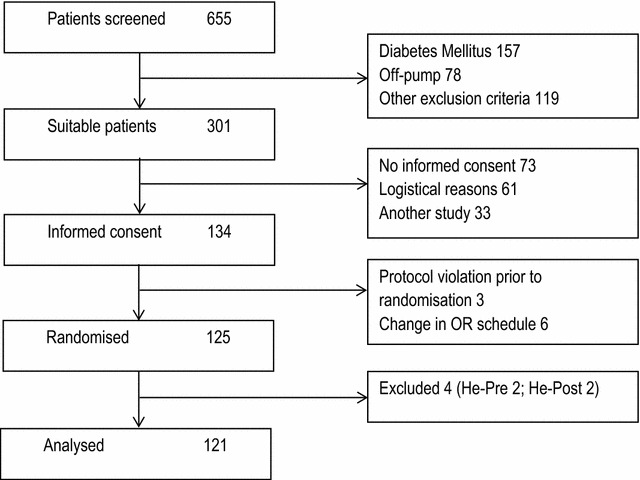
CONSORT diagram

Four patients were excluded from the study (no helium available (1), no investigational team available (1), unplanned additional valve surgery (1), previously unknown decreased left ventricular ejection fraction <30 % (1)), leaving data from 121 patients available for statistical analysis (CONSORT diagram see Fig. 2). Baseline characteristics of all groups were similar with regard to age (66 ± 9) and sex (83 % male, Table 1). Preoperative demographic data showed that in controls, less patients with hypercholesterolemia, or who used statins or beta-blockers were present compared to the other groups. The predictive additive risk determined by Euroscore was similar in all groups. The number of bypass grafts and duration of aortic cross clamping and CPB duration were also similar in all groups (Table 2).

**Table 1:** Demographic data

	Controls	He-pre	He-post	He-PP	APC	*p * value
N	28	23	22	24	24	
Age ± years	66.7 ± 7.0	62.8 ± 11.7	66.2 ± 7.8	66.2 ± 8.3	66.9 ± 7.6	*0.47*
Male (%)	22 (82)	21 (91)	20 (83)	20 (83)	21 (88)	*0.88*
Body Mass Index	28.4 ± 3.7	27.3 ± 2.7	26.7 ± 3.8	27.4 ± 3.7	27.7 ± 3.2	*0.55*
Risk factors						
Hypertension	16 (59)	9 (39)	13 (57)	10 (42)	13 (54)	*0.52*
Hypercholesterolemia	6 (22)	11 (48)	10 (44)	5 (21)	14 (58)	*0.023* ^a^
Smoking	13 (48)	10 (44)	8 (34)	11 (46)	10 (42)	*0.89*
Family	12 (44)	9 (39)	7 (30)	9 (38)	13 (54)	*0.56*
Myocardial infarction	7 (26)	10 (44)	8 (36)	10 (42)	8 (33)	*0.71*
Cerebrovascular accident	0 (0)	0 (0)	0 (0)	1 (4)	0 (0)	*0.40*
Heart failure						
NYHA I	20 (74)	17 (74)	19 (86)	17 (71)	16 (70)	*0.97*
NYHA II	2 (7)	2 (9)	1 (5)	2 (8)	3 (13)	*0.97*
NYHA III	5 (19)	4 (17)	2 (9)	4 (17)	4 (17)	*0.97*
Ejection fraction						
>50 %	21 (81)	16 (73)	12 (57)	16 (67)	19 (90)	*0.15*
30–50 %	5 (19)	6 (27)	7 (33)	4 (17)	1 (5)	*0.15*
Medication						
Salicylate	24 (86)	19 (83)	22 (92)	23 (96)	23 (92)	*0.57*
Clopidogrel	9 (32)	9 (39)	12 (50)	9 (38)	4 (16)	*0.15*
Statin^b^	27 (96)	23 (100)	24 (100)	20 (83)	21 (84)	*0.04* ^b^
Beta-blocker^c^	19 (68)	22 (96)	24 (100)	21 (88)	21 (84)	*0.008* ^c^
ACE inhibitor	10 (36)	8 (35)	10 (42)	4 (17)	11 (44)	*0.30*
AT2 receptor blocker	6 (21)	1 (4)	5 (21)	5 (21)	3 (12)	*0.40*
Calcium channel blocker	10 (36)	7 (30)	4 (17)	10 (42)	6 (24)	*0.35*
Diuretics	6 (21)	4 (17)	4 (17)	7 (29)	4 (16)	*0.77*
Nitrates	10 (36)	9 (39)	7 (29)	12 (50)	13 (52)	*0.43*
Euroscore	3 (±2)	2 (±3)	3 (±3)	4 (±3)	1 (±3)	*0.36*

Age and body mass index are presented as mean ± SD; Euroscore is presented as mean (±IQR); other data are numbers and percentage, n (%)

^a^In the control group significantly less patients with hypercholesterolemia were present

^b^Use of statins was significantly lower in our control group compared to other groups

^c^Use of beta-blockers was significantly lower in our control group compared to other groups

*He-Pre* helium preconditioning; *He-Post* helium postconditioning; *He-PP* helium pre- and postconditioning; *APC* anesthetic preconditioning

Age and Body Mass Index are presented as mean ± SD; Euroscore is presented as mean (±IQR); other data are numbers and percentage, n (%). In the control group significantly less patients with hypercholesterolemia were present. b Use of statins was significantly lower in our control group compared to other groups. c Use of beta-blockers was significantly lower in our control group compared to other groups

**Table 2:** Surgical specifications

	Controls	He-pre	He-post	He-PP	APC	*p * value
ECC time (min)	88 ± 7	84 ± 6	91 ± 5	100 ± 7	95 ± 6	*0.53*
Cross-clamp time (min)	59 ± 4	54 ± 4	58 ± 5	67 ± 5	62 ± 4	*0.51*
Cardioplegia						
Blood (%)	18 (82)	17 (85)	18 (82)	14 (67)	18 (78)	*0.36*
Saline (%)	4 (18)	3 (15)	4 (18)	7 (33)	5 (22)	*0.36*
Number of coronary arteries						
1	1 (4)	1 (5)	0 (0)	2 (8)	1 (4)	*0.19*
2	9 (33)	6 (27)	4 (17)	8 (33)	7 (29)	*0.19*
3	17 (63)	15 (68)	17 (81)	14 (58)	16 (67)	*0.19*
Left main	9 (32)	8 (36)	3 (13)	20 (42)	10 (40)	*0.88*

Data are presented as mean ± SD; no significant differences were observed between groups

*He-Pre* helium preconditioning; *He-Post* helium postconditioning; *He-PP* helium pre- and postconditioning; *APC* anesthetic preconditioning

### Pre- and postconditioning protocols

Helium and sevoflurane pre- and/or postconditioning was administered without any problems during surgery. Neither the preconditioning nor the postconditioning stimulus altered hemodynamics, and no differences in heart rate, mean arterial pressure, cardiac index or pulmonary artery pressure were observed between groups (Table 3). We did not measure myocardial oxygen supply directly, but no differences were observed between groups regarding arterial oxygen tension and hemoglobin levels (see Additional file 2).

**Table 3:** Hemodynamic data

	Controls	He-pre	He-post	He-PP	APC
Heart rate (beats min^−1^)					
After sternotomy	56 ± 7	59 ± 8	62 ± 12	60 ± 10	60 ± 13
After preconditioning	60 ± 11	59 ± 8	59 ± 9	61 ± 11	61 ± 13
After CPB	72 ± 7	73 ± 10	73 ± 14	71 ± 12	80 ± 15
Mean arterial pressure (mmHg)					
After sternotomy	76 ± 12	74 ± 13	76 ± 14	77 ± 13	80 ± 16
After preconditioning	66 ± 12	69 ± 13	64 ± 12	67 ± 11	68 ± 13
After CPB	63 ± 12	64 ± 7	66 ± 11	70 ± 11	70 ± 14
Cardiac Index (L/min/m^2^)					
After sternotomy	2.1 ± 0.6	2.1 ± 0.6	2.0 ± 0.4	2.1 ± 0.4	2.2 ± 0.5
After preconditioning	2.0 ± 0.7	2.1 ± 0.5	2.0 ± 0.4	2.3 ± 0.4	2.1 ± 0.5
After CPB	2.4 ± 0.7	2.7 ± 0.8	2.4 ± 0.4	2.5 ± 0.7	2.1 ± 0.8
Pulmonary artery pressure (mmHg)					
After sternotomy	18 ± 6	16 ± 5	16 ± 5	17 ± 4	20 ± 3
After preconditioning	18 ± 6	18 ± 6	14 ± 5	17 ± 4	18 ± 5
After CPB	20 ± 6	18 ± 4	18 ± 4	20 ± 5	20 ± 4
PCWP (mmHg)					
After sternotomy	11 ± 6	10 ± 6	8 ± 6	10 ± 2	8 ± 4
After preconditioning	4 ± 5	9 ± 6	9 ± 7	9 ± 3	9 ± 5
After CPB	15 ± 6	11 ± 3	8 ± 3	13 ± 3	10 ± 1

Data are presented as mean ± SD; no significant differences were observed between groups

*He-Pre* helium preconditioning; *He-Post* helium postconditioning; *He-PP* helium pre- and postconditioning; *APC* anesthetic preconditioning; *CPB* cardiopulmonary bypass; *PCWP* pulmonary capillary wedge pressure

### Phosphorylation of p38 MAPK in cytosolic fraction

**Fig. 3:**
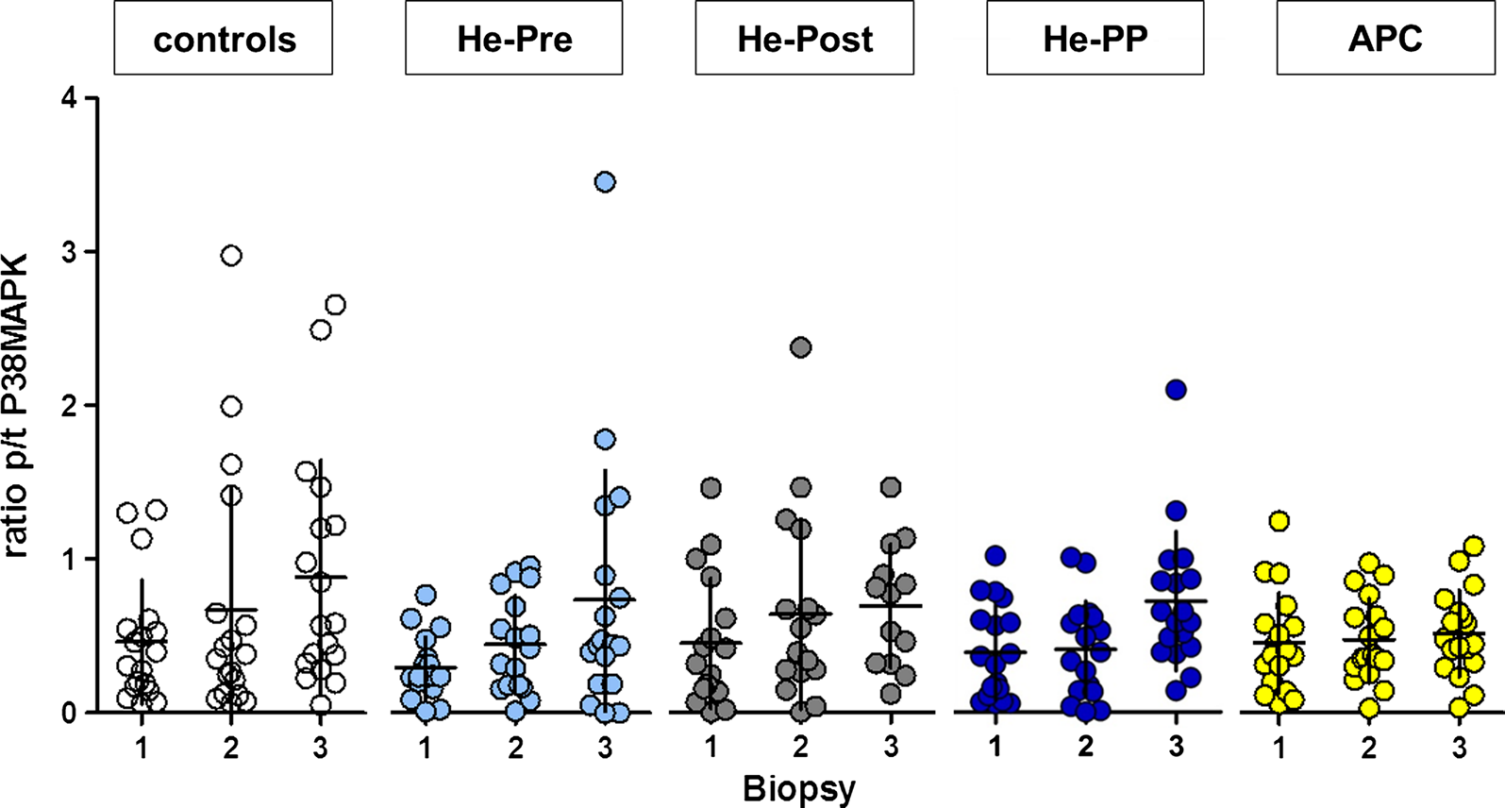
Ratio of activated p38 MAPK. The ratio of activated (phosphorylated to total p/t p38MAPK) at three different time points. The baseline ratio p/t p38MAPK is represented by biopsy 1. Biopsy 2 represents the ratio directly after application of the preconditioning stimulus, and the biopsy 3 is at the end of cardiopulmonary bypass. Values presented in mean ± SD. No significant differences were observed in the ratios of p/t p38MAPK at different time points in the controls (*white dots*), after preconditioning with helium (He-Pre; *light blue dots*) or sevoflurane (APC; *orange dots*), or after postconditioning (He-Post; *grey dots*) or the combination of helium pre- and postconditioning (He-PP; *dark blue dots*, Kruskall Wallis rank sum analysis)

We determined in the cytosolic fraction of the myocardial atrial tissue the phosphorylated-to-total (p/t) p38 MAPK ratios within three biopsies from each patient taken at time points described in Fig. 3. In controls, no statistically significant changes were observed at begin of CPB and after CPB compared to baseline (Fig. 3). In groups receiving a preconditioning stimulus (He-Pre, He-PP, APC), no effect of the preconditioning stimulus on the ratio of p/t p38 MAPK was observed (biopsy 2 vs. biopsy 1). In addition, also postconditioning (He-Post, He-PP) did not affect the ratio p/t p38 MAPK (biopsy 3 vs. biopsy 1).

### Phosphorylation of ERK-1 and ERK-2 in cytosolic fraction

**Fig. 4:**
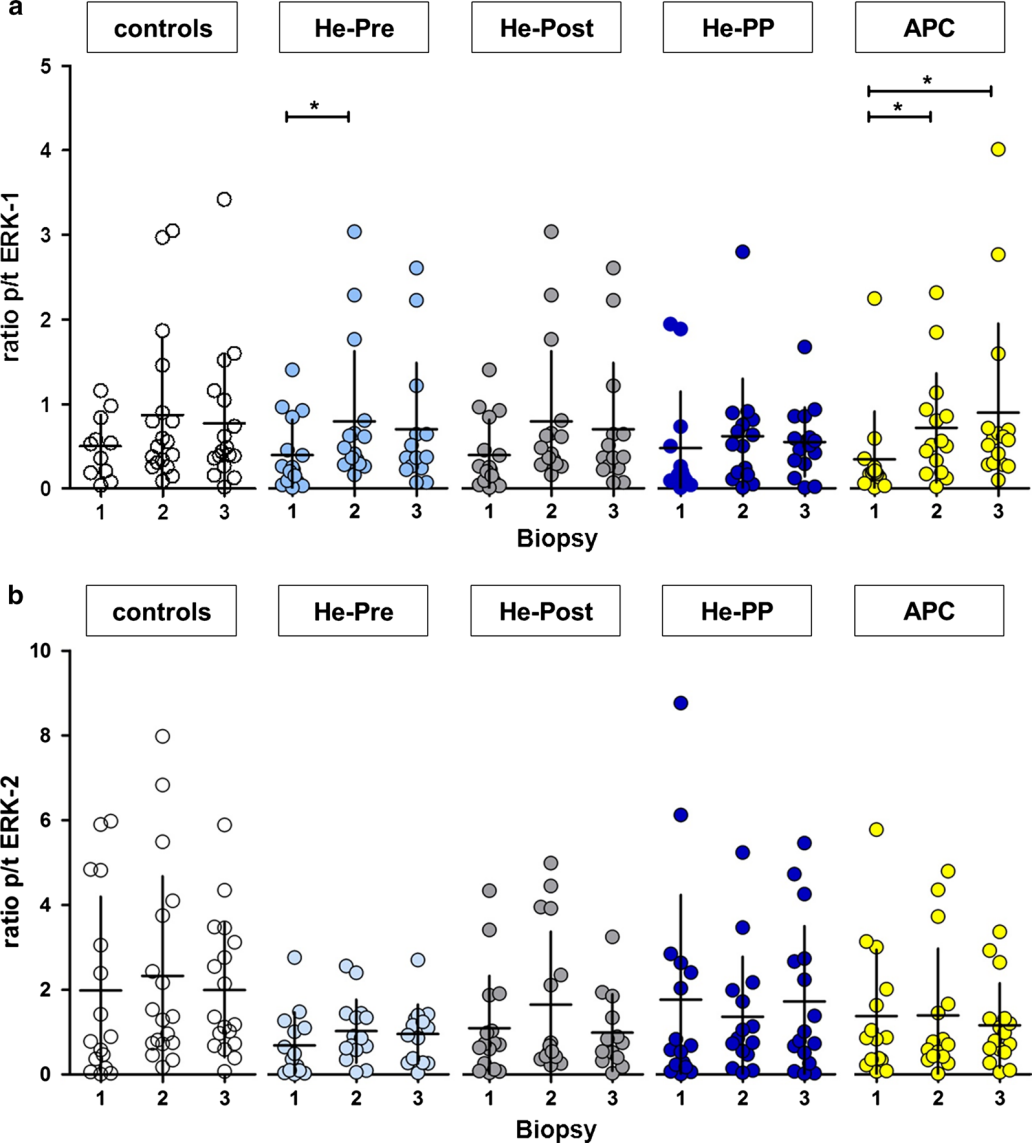
Ratio of activated ERK-1 and ERK-2. Ratio of activated (phosphorylated to total, p/t) Extracellular-signal regulated kinases-1 and -2 at different time points. **a** ERK-1 (p44) and **b** ERK-2 (p42). *X axis* presents ratio of p/t ERK-1 or -2, *Y axis* represents different biopsies (1–3) for each group (see also legend Fig. 3). Values presented in mean ± SD. For ERK-1, we observed a significant increase in p/t ratio in the helium-preconditioning group in the second biopsy (taken after preconditioning) compared to baseline. In the anaesthetic preconditioning group, we observed a significant increase compared to controls for biopsy 2 and 3. No significant differences in the ratio of p/t ERK-2 were observed at the different time points

The ratio of activated (phosphorylated to total, p/t) ERK-1 of the cytosolic fraction was determined. In the preconditioning groups (He-Pre, APC), a statistically significant increase of ratio p/t ERK-1 was observed after the preconditioning stimulus (biopsy 2 vs. biopsy 1; Fig. 4 upper panel). While these changes were still evident after CPB in the APC group, the changes were no longer significantly different from baseline at the end of CPB in the He-Pre group. Postconditioning with helium had no effect on ratio p/t ERK-1.

Regarding the ratio p/t ERK-2, no statistically significant changes were observed after either pre- or postconditioning with helium or sevoflurane, nor in the control group (Fig. 4, lower panel).

### Protein expression of HSP-27 in particulate fraction

**Fig. 5:**
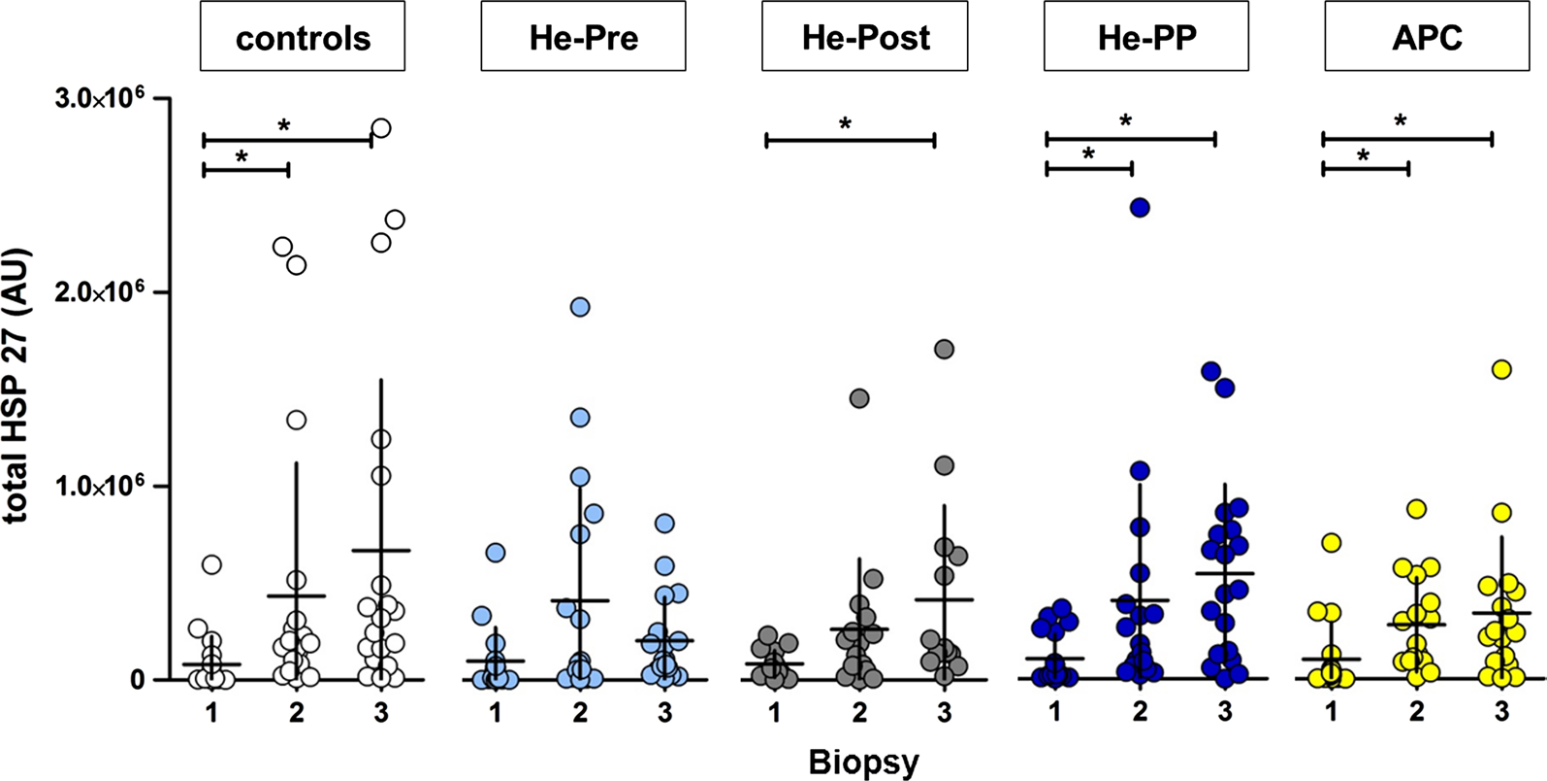
Levels of HSP-27. Amount of heat shock protein (HSP)-27 (mean ± SD) of the particulate fraction at different time points; *X axis* is net intensity of the signal of HSP-27. *Y axis* represents different biopsies (1–3) for each group (see also legend Fig. 3). Except for the helium-preconditioning group, a significant increase of total HSP-27 at the end of cardiopulmonary bypass (biopsy 3) compared to the respective baseline values was found. For the controls, helium pre- and postconditioning group and the anesthetic preconditioning group this increase was also significant directly after preconditioning (biopsy 2) compared to baseline

Total levels of HSP-27 were determined in the particulate fraction of myocardial atrial tissue. In controls, we observed a statistically significant increase of HSP-27 immediately before as well as after CPB (biopsy 2 and 3 vs. biopsy 1, respectively; Fig. 5). The same pattern of changes in HSP-27 as observed in the control group was seen in all other pre- and postconditioning groups, with the only exception that in the He-Pre group no increase of HSP-27 after CPB (biopsy 3) was seen.

### Expression of PKC-ε in the particulate fraction

**Fig. 6:**
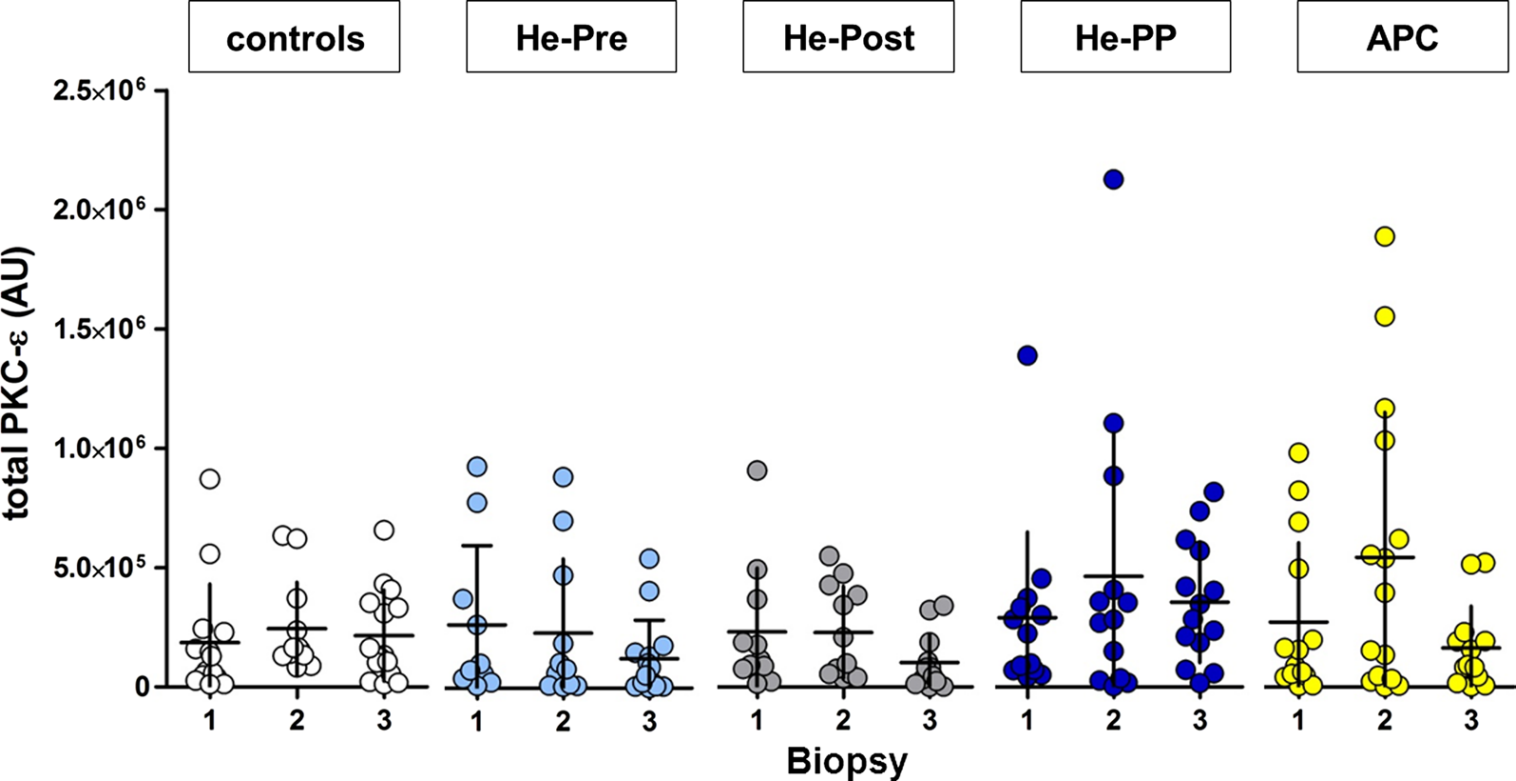
Levels of PKC-ε in particulate fraction. Levels of protein kinase C-ε (PKC-ε) in particulate fraction of myocardial atrial tissue at different time points (mean ± SD, see also legend Fig. 3). No differences of PKC-ε were observed at different time points in controls, after preconditioning with helium (He-pre) of sevoflurane (APC) or after helium postconditioning (He-post) or the combination of helium pre-and postconditioning (He-PP)

Total PKC-ε was determined in the particulate fraction of the atrial tissue. In control patients, we did not observe any statistically significant difference of PKC-ε levels immediately before or after CPB compared to baseline (biopsy 2 and 3 vs. biopsy 1, respectively; Fig. 6). Neither preconditioning with helium or sevoflurane (He-Pre, He-PP, APC), nor postconditioning with helium (He-PP, He-Post) had any statistically significant effect on levels of PKC-ε before or after CPB.

### Plasma concentrations of postoperative troponin T

**Fig. 7:**
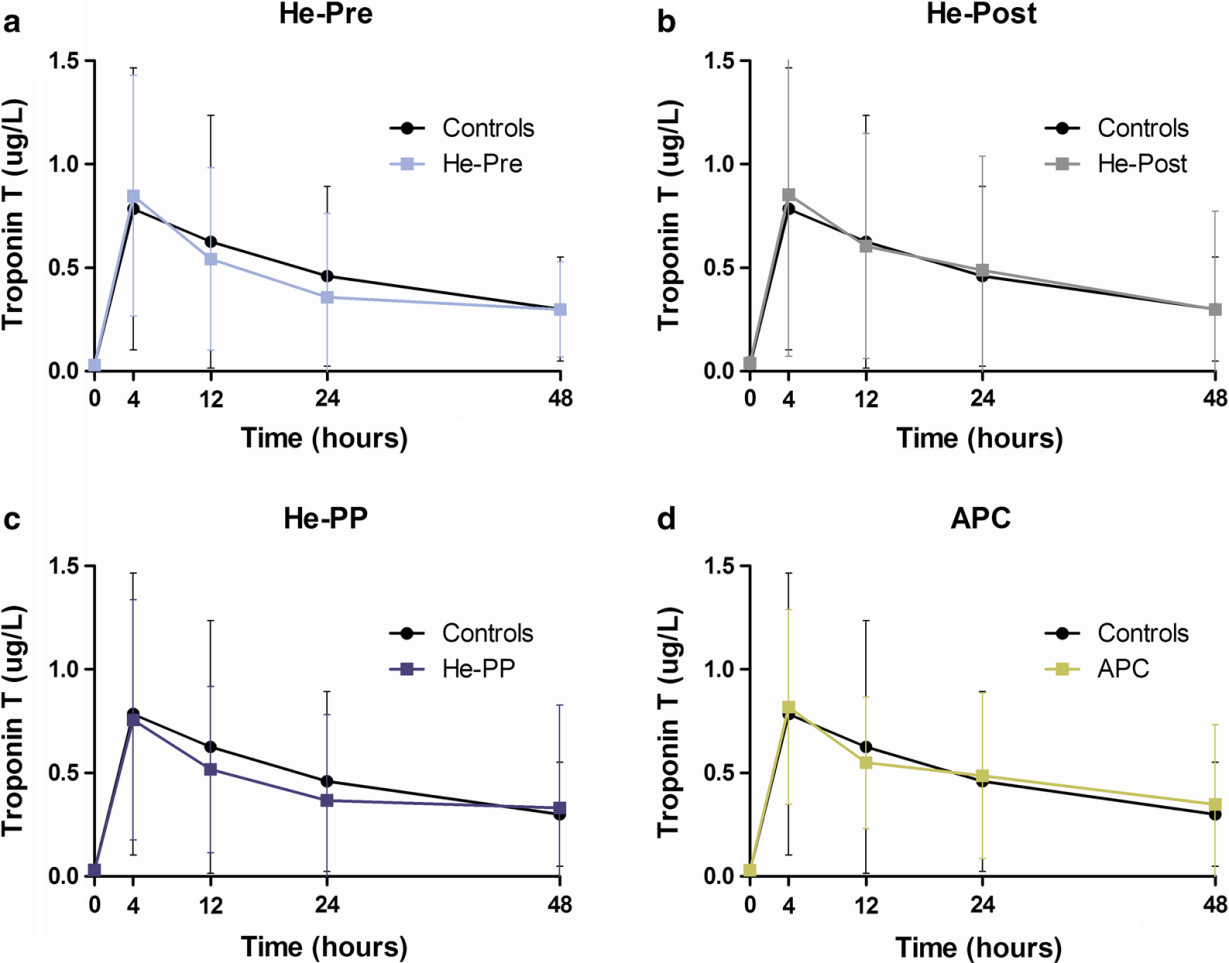
Postoperative troponin T levels. The course of postoperative troponin T release; all groups compared to controls (represented in *black* in all *panels*). Helium preconditioning (He-pre) does not affect postoperative troponin T levels compared to controls (**a**), nor does helium postconditioning (He-post, **b**) or the combination of helium pre- and postconditioning (He-PP, **c**). Anesthetic preconditioning (APC) with sevoflurane did also not affect postoperative troponin T levels compared to controls in the present study (**d**). *X axis* represents troponin T level in μg/L, values in median ± interquartile range. *Y axis* represents time (hours)

Postoperative troponin T was 11 arbitrary units [5, 31; area-under-the-curve (interquartile range)] for the controls, and no statistically significant changes were observed after helium preconditioning [He-Pre: 11 (6, 18)], helium postconditioning [He-Post: 11 (8, 15)], helium pre- and postconditioning [He-PP: 14 (6, 20)] and after sevoflurane preconditioning [APC: 12 (8, 24), p = 0.13, one-way ANOVA after log transformation, Fig. 7].

## Discussion

In the present study, we did not observe any relevant differences on a molecular level in the regulation patterns of the signal transduction kinases p38 MAPK, ERK-1, ERK-2, or PKC-ε after helium treatment as compared to the control group. He preconditioning alone (without postconditioning) prevented HSP-27 increase as observed in all other groups. Helium pre- and postconditioning—alone or in combination—did not affect postoperative troponin T release in patients undergoing CABG surgery.

### Molecular changes by helium

Mechanisms underlying the protection by volatile anesthetics and noble gases have been investigated extensively in animal experiments [8, 21, 22]. However, mechanistic data from human studies are scarce. We investigated whether helium has any influence on signal transduction markers known to play a role in noble gas induced cardioprotection we demonstrated before [3–5, 7, 23]. Although p38 MAPK plays a role in inhalational anesthetic induced preconditioning [4], we were unable to demonstrate a role for p38 MAPK in preconditioning with either helium or sevoflurane in this clinical study: the ratio of phosphorylated to total p38 MAPK in the cytosolic fraction of the myocardial atrial tissue showed no statistically significant differences at various time-points in all groups. In contrast, Pouzet et al. demonstrated an increase of p38 MAPK after CPB in controls and sevoflurane treated patients undergoing CABG surgery [24]. No statistically significant difference in PKC-ε levels after preconditioning with helium, sevoflurane or in untreated controls was observed, which is in contrast to a previous study showing translocation of PKC-ε to the particulate fraction after sevoflurane preconditioning [19].

The ratio of phosphorylated-to-total ERK-1 was increased after helium preconditioning compared to the baseline value, but this effect was no longer present after reperfusion at the end of CPB. In contrast, the increased ratio of phosphorylated-to-total ERK-1 after sevoflurane preconditioning was still present at the end of CPB. For ERK-2, no statistically significant effects were found at any time-point in any of the groups. In contrast, Talmor et al. demonstrated in atrial tissue obtained at similar time-points during CABG surgery an increase in ERK-1/2 activity after ischemia and reperfusion [25].

Several studies investigated changes of HSP-27 during cardiac surgery, most of them measuring HSP in patient blood [26–28]. Our data demonstrate that in untreated controls the level of HSP-27 in atrial tissue increases significantly after reperfusion compared to baseline levels. Except for the helium-preconditioning group, this effect was seen in all treatment groups, indicating that aortic cross clamping and subsequent reperfusion increases the levels of HSP-27.

Besides the data on PKC-ε, which were used for the power calculation, there were no data available of ERK1/2, HSP-27 or p38MAPK in human myocardial tissue after preconditioning at start of the current study. Although unlikely, we cannot completely exclude the possibility that our study was underpowered to detect a difference in ERK1/2, HSP-27 or p38MAPK. A clinically relevant outcome parameter to be alternatively used for power calculation would have been troponin T values during the postoperative course. This parameter was used in a previous study with much smaller groups sizes (n = 10) [16], showing a significant difference between groups. Therefore, we expected to find any clinical relevant differences of troponin release with the current group size of 25 patients per group.

### Lack of sevoflurane preconditioning

In the present study we were unable to show protection in the suggested positive control, namely sevoflurane preconditioning. Demographic data, duration of CPB and aortic cross clamping did not differ compared to recent studies showing cardioprotection by sevoflurane [18, 19].

In our previous study [19], all patients received crystalloid cardioplegia, while in the current study crystalloid as well as blood cardioplegia was allowed. However, the distribution of crystalloid and blood cardioplegia was not significantly different between groups. Theoretically, a diminished ischemic burden could have affected the power needed to obtain protection. Only two cardiac surgeons performed all procedures in the previous study, while in the current study numerous surgeons were involved. Larger than expected variations in biopsies, both in size as in composition (percentage of muscle and fatty tissue), might also have influenced our molecular results. More detailed information regarding difficulties we encountered during protein analysis of these samples are described in Additional file 3. Whether these increased diversities in clinical practices might have blunted potential cardioprotective effects of sevoflurane preconditioning remains unclear.

Opioid-induced cardioprotection might affect additional cardioprotection by inhalational agents [29], however all patients received opioids in a comparable dosage, which was also performed in our previous study, and it is unlikely that this has significantly influenced the results.

Surprisingly, we did not observe any cardioprotective effect as measured by troponin T release (see Fig. 7). The volatile anesthetic sevoflurane is one of the few preconditioning agents that so far was successfully translated from experimental studies into clinical practice: sevoflurane reduced postoperative troponin release after CABG surgery [17–19]. Several meta-analysis showed that the modern volatile anesthetics sevoflurane and desflurane were associated with a reduction in mortality after cardiac surgery when compared with total intravenous anesthesia [30, 31]. The original data from the studies included in these reviews contain small patient groups, and the studies used different conditioning protocols and stimuli. Another review, focussing on the preconditioning protocol used [32], mentioned that protection could be a side effect of sevoflurane induced alterations in myocardial oxygen demand and supply, not necessarily indicating preconditioning. Despite the initial successful translation of anesthetic preconditioning into clinical practice, more recent studies show more variable or even contradictory results. For a definitive answer on whether sevoflurane induces preconditioning and which modality of its application is most effective, larger randomised controlled trials are needed to provide more robust evidence.

### Lack of helium pre- and postconditioning

While the stimulus for preconditioning is applied before myocardial ischemia, postconditioning is the protection induced by a stimulus applied during ischemia or at the beginning of reperfusion. Presence of the stimulus during reperfusion seems to be essential for its success to evoke protection. *Ischemic* postconditioning decreased postoperative troponin release after cardiac surgery in children, [33] and decreased postoperative CK-MB but not troponin I release in adults [34].

Helium induces protection by postconditioning [13], but the current results do not show a beneficial effect of helium postconditioning. We started helium postconditioning at the end of aortic cross clamping by manual ventilation of the lungs—while the patient was still on CPB. We did not measure coronary artery (collateral) flow, nor did we measure helium concentration in coronary blood. We therefore cannot confirm that sufficient helium was present within the coronary artery system at the beginning of cardiac reperfusion, and it is possible the postconditioning stimulus was insufficient. We used 70 % helium, allowing an inspiratory concentration of oxygen of 30 %. Although experimental data indicate a concentration of 30–70 % helium to be enough to induce preconditioning [14], it cannot be excluded that 70 % helium was too low to induce protection in CABG patients with increased age and multiple comorbid conditions. We previously showed helium induced preconditioning was abolished in aged [35] as well as in hypertensive animals [36]. However, in the hypertensive rat, a combination of helium induced pre- and postconditioning was able to overcome the barrier for cardioprotection, leading to reduction of infarct size [36]. In our current study, thirteen patients (57 %) from the He-PP group had hypertension. However, even the combination of helium pre- and postconditioning did not result in reduction of troponin release in this patient group.

It could be that the general trauma for CABG has reduced over time and therefore it will become more and more difficult for a protecting agent to show an additional benefit. Most likely, the most “healthy” CABG patients will not profit from additional protection, and the possible protective effects of helium in high-risk cardiac surgery patients (e.g., valve-plus-CABG surgery, thoracic aortic surgery) are still unknown.

## Conclusion

In patients subjected to on-pump CABG surgery, we could not observe any statistically significant effect of helium on enzymes of the signal transduction cascade of pre- and postconditioning in human atrial tissue, or on troponin T release. The use of helium as a cardioprotective agent is still a matter of debate between different study groups; however, this is not the case with sevoflurane, which was also without activity in the current study, but brings into question the robustness and true translational value of this type of cardioprotection in CABG surgery.

## Additional files

10.1186/s12967-016-1045-z Mean differences of western blot data.

10.1186/s12967-016-1045-z Table of perioperative oxygen tension and hemoglobin.

10.1186/s12967-016-1045-z Technical problems using human atrial tissue for protein detection with western blot.

10.1186/s12967-016-1045-z Raw data of the western blots.

10.1186/s12967-016-1045-z Raw data of the troponin levels.

## Abbreviations

APC: anesthetic preconditioning
CABG: coronary artery bypass grafting
CPB: cardiopulmonary bypass
ERK1/2: extracellular signal-regulated kinase 1/2
He-Post: helium postconditioning
He-PP: helium pre- and postconditioning
He-Pre: helium preconditioning
HSP-27: heat shock protein 27
p38 MAPK: mitogen activated protein kinase 38
MAC: minimal alveolar concentration
PKC-ε: protein kinase C-epsilon
TBS-T: tris-buffered saline containing tween
