# Supplementary material for: Effect of helium pre- or postconditioning on signal transduction kinases in patients undergoing coronary artery bypass graft surgery
Source: J Transl Med. 2016 Oct 14;14:294. doi: 10.1186/s12967-016-1045-z (PMC5064802; doi:10.1186/s12967-016-1045-z)
Supplement: Supplementary file 1 — 10.1186/s12967-016-1045-z Mean differences of western blot data. [file 12967_2016_1045_MOESM1_ESM.doc]

**Additional file 1: mean differences of western blot data**

**-Table 1:** mean differences of p38 Mitogen Activated Protein Kinase

|  | **Mean difference (95% confidence interval)** | |
| --- | --- | --- |
|  | **Time 1-2** | **Time 1-3** |
| Control | 0.21 (-0.13 to 0.54) | 0.42 (0.07 to 0.77) |
| He-Pre | 0.15 (-0.01 to 0.31) | 0.44 (0.02 to 0.86) |
| He-Post | -0.19 (0.04 to 0.34) | 0.34 (0.09 to 0.59) |
| He-PP | 1.35 (-4.33 to 1.62) | -1.91 (-6.63 to 2.80) |
| APC | 0.02 (-0.18 to 0.21) | 0.06 (-0.13 to 0.25) |

**-Table 2:** mean differences extracellular regulated kinase -1

|  | **Mean difference (95% confidence interval)** | |
| --- | --- | --- |
|  | **Time 1-2** | **Time 1-3** |
| Control | 0.62 (-0.04 to 1.28) | 0.56 (0.05 to 1.08) |
| He-Pre | 0.17 (0.02 to 0.31) | 0.13 (-0.05 to 0.31) |
| He-Post | 0.40 (-0.01 to 0.80) | 0.33 (-0.22 to 0.88) |
| He-PP | 0.35 (-1.98 to 0.90) | 0.19 (-0.13 to 0.51) |
| APC * | 0.31 (0.13 to 0.50) | 0.35 (-0.13 to 0.51) |

**-Table 3:** mean differences extracellular regulated kinase -2

|  | **Mean difference (95% confidence interval)** | |
| --- | --- | --- |
|  | **Time 1-2** | **Time 1-3** |
| Control | 0.57 (-0.46 to 1.59) | 0.18 (-0.70 to 1.05) |
| He-Pre | 0.39 (-0.04 to 0.81) | 0.26 (-0.23 to 0.75) |
| He-Post | 0.58 (-0.25 to 1.41) | -0.008 (-0.58 to 0.57) |
| He-PP | 0.26 (-0.19 to 0.71) | 0.11 (-0.83 to 1.05) |
| APC | 0.09 (-0.27 to 0.45) | 0.05 (-0.24 to 0.34) |

**-Table 4:** mean differences heat shock protein 27

|  | **Mean difference (95% confidence interval)** | |
| --- | --- | --- |
|  | **Time 1-2** | **Time 1-3** |
| Control | 351923 (54200 to 649648) | 587276 (195040 to9795120 |
| He-Pre | 303491 (1571 to 605412) | 112993 (-13115 to 239101) |
| He-Post | 178773 (-1678 to 359224) | 331187 (66224 to 596151) |
| He-PP | 294947 (-15916 to 605810) | 420241 (225811 to 614670) |
| APC | 233016 (74261 to 391773) | 323358 (97583 to 549133) |

**-Table 5:** mean differences protein kinase C-ε

|  | **Mean difference (95% confidence interval)** | |
| --- | --- | --- |
|  | **Time 1-2** | **Time 1-3** |
| Control | 44292 (-89173 to 177757) | 42817 (-53249 to 138883) |
| He-Pre | 184134 (-90656 to 458925) | -166053 (-343517 to 11411) |
| He-Post | -23844 (-148205 to 100516) | -91881 (-330778 to 147016) |
| He-PP | 136673 (-311139 to 584486) | 95976 (-70820 to 26773) |
| APC | 331781 (50277 to 613285) | -58411 (-217277 to 100455) |

He-Pre: helium preconditioning; He-Post: helium postconditioning; He-PP: helium pre- and postconditioning; APC: anaesthetic preconditioning with sevoflurane
