# Supplementary material for: Effect of helium pre- or postconditioning on signal transduction kinases in patients undergoing coronary artery bypass graft surgery
Source: J Transl Med. 2016 Oct 14;14:294. doi: 10.1186/s12967-016-1045-z (PMC5064802; doi:10.1186/s12967-016-1045-z)
Supplement: Supplementary file 2 — 10.1186/s12967-016-1045-z Table of perioperative oxygen tension and hemoglobin. [file 12967_2016_1045_MOESM2_ESM.doc]

**Additional file 2: Table of peroperative oxygen tension and hemoglobin**

|  |  | **Controls** | **He-Pre** | **He-Post** | **He-PP** | **APC** |
| --- | --- | --- | --- | --- | --- | --- |
| **Hemoglobin** (mmol/L) | |  |  |  |  |  |
|  | *Before CPB* | 5,7±0,6 | 5,7±0,8 | 5,4±0,7 | 5,4±0,8 | 5,9±1,0 |
|  | *after CPB* | 6,0±0,6 | 5,9±1,0 | 5,6±0,8 | 5,6±0,8 | 6,2±0,7 |
| **Oxygen tension** (kPa) | |  |  |  |  |  |
|  | *Before CPB* | 23±7 | 21±8 | 21±7 | 21±8 | 19±7 |
|  | *after CPB* | 21±14 | 23±19 | 18±7 | 23±19 | 20±13 |

Data are presented as mean ± SD; no significant differences were observed between groups.

He-Pre= Helium preconditioning; He-Post=Helium Postconditioning; He-PP= Helium Pre- and Postconditioning; APC=Anesthetic preconditioning.
