# Supplementary material for: Effect of helium pre- or postconditioning on signal transduction kinases in patients undergoing coronary artery bypass graft surgery
Source: J Transl Med. 2016 Oct 14;14:294. doi: 10.1186/s12967-016-1045-z (PMC5064802; doi:10.1186/s12967-016-1045-z)
Supplement: Supplementary file 3 — 10.1186/s12967-016-1045-z Technical problems using human atrial tissue for protein detection with western blot. [file 12967_2016_1045_MOESM3_ESM.doc]

Online supplement 3: Technical problems using human atrial tissue for protein detection with western blot

Our first problem was the difference in size of the atrial biopsies that were taken by different surgeons. This problem was solved during the time of the study, as both the surgeons and the investigators became more experienced. However, even with the sizes of the biopsies similar, the content and more specifically the amount of fat tissue involved, varied significantly among patients. This resulted in great variations of protein content of the samples after preparation for Western blotting. As all the samples were standardized to protein content for western blotting, standardizing was more difficult with varying concentrations. For some of the samples with low protein concentrations, the amount of sample for Western Blot was low, allowing us only to perform a few blots.

The western blot method itself was not different with human tissue, but the antibody concentration needed for quantifiable bands was different from experiments in tissues from other species. We needed several blots to establish steady and quantifiable results. This involved switching back from our infrared odyssey imager to chemiluminescence detection on X-ray film.

After establishing a sound method for analysis of these four targets (P38 MAPK, ERK1/2, HSP27 and PKC- ε) we changed to western blot system using precast gels and a larger western blot tank, allowing more samples to be analyzed at the same time. However, establishing these methods left us with partly insufficient sample for analysis with our final blotting system.
